# Associations between HIV infection status, psychosocial factors, and adjuvant endocrine therapy adherence among South African women with early-stage breast cancer

**DOI:** 10.21203/rs.3.rs-4559587/v1

**Published:** 2024-06-27

**Authors:** Daniel S O’Neil, Charmaine L Blanchard, Maureen Joffe, Michael Antoni, Molly Ream, Keletso Constance Mmoledi, Nontlantla Mkwanazi, Victor Shandukani, Paul Ruff

**Affiliations:** Yale University; University of Witwatersrand; University of Witwatersrand; University of Miami; University of Miami; University of Witwatersrand; University of Witwatersrand; University of Witwatersrand; University of Witwatersrand

**Keywords:** Breast cancer, HIV, Adherence, South Africa, Endocrine therapy

## Abstract

**Purpose:**

We aimed to evaluate for associations between HIV status, psychosocial factors, and adjuvant endocrine therapy (AET) adherence in South African (SA) women with estrogen receptor positive (ER+) breast cancer (BC).

**Methods:**

We enrolled South African women with early-stage ER + BC in remission and prescribed tamoxifen or an aromatase inhibitor to the prospective observational study. We performed AET pill counts at enrollment, 12 weeks, and 24 weeks, and calculated adherence ratios of pills consumed between visits to days between visits. Women completed questionnaires on social support, attitude towards medication, health literacy, self-efficacy, mental health, and AET toxicity. We collected household wealth data. We used hierarchical linear (HLM) and structural equation modelling (SEM) to compare adherence ratios between women with and without HIV while adjusting for psychosocial factors.

**Results:**

We collected adherence data from 239 women, 63 (26.4%) with co-morbid HIV. Comparing women with and without HIV, median AET adherence ratio was 0.88 vs 0.89, respectively (HLM p = 0.31). In our SEM model for the full cohort, mental health, healthcare savvy, and side effect burden latent variables were not significantly associated with adherence. In the subgroup of women living with HIV, lower SES quintile (β 0.04, SE 0.02, p = 0.08) and poorer mental health (β −0.02, SE 0.01, p = 0.10) showed trends toward association with adherence.

**Conclusions:**

HIV status is not predictive of AET adherence among SA women with ER + BC, though decreasing SES status and increasing mental health symptoms are marginally associated with adherence in women with BC and HIV.

## Introduction

Analyses of the National Cancer Database, SEER-Medicare, and the US-based HIV/AIDS Cancer Match Study registries demonstrate that breast cancer (BC) patients living with comorbid HIV experience increased cancer-specific mortality (hazard ratios (HRs) from 1.85–2.64) and overall mortality (HR 1.85) compared to other BC patients.[1–3] The South African Breast Cancer and HIV Outcomes (SABCHO) cohort study found similar survival disparities among stage I-III South African BC patients with HIV (HR 1.49).[4] Unfortunately, 20% of new BC diagnoses in South Africa’s (SA) public healthcare system are in women living with HIV (WLWH).[5, 6] Understanding the cause of BC survival disparities in WLWH is necessary for improving outcomes in this vulnerable population.

Adjuvant endocrine therapy (AET) for early, hormone receptor positive (HR+) BC, reduces cancer recurrence by half and mortality by a third over fifteen years.[7, 8] Clinical trial and real world data show increased mortality in BC survivors who prematurely stop adjuvant endocrine therapy (AET), yet poor adherence to AET is seen in ~ 50% of HR + BC survivors in high-income countries (HICs) due to medication side effects, psychologic distress, distrust of chronic medications, and other factors.[7, 9–16] Factors associated with non-adherence in low- and middle-income countries (LMICs), including SA, are understudied, yet patients are at high risk for factors known to contribute to non-adherence. The 20% of BC survivors living with HIV may be especially vulnerable to toxicity or distress from polypharmacy and dual diagnoses, but the relationship between AET adherence and comorbid HIV has not been widely studied. Our own early work in Black South African women with early stage breast cancer has shown lower serum concentrations of tamoxifen in participants with comorbid HIV.[17]

With this prospective observational study, we aimed to further characterize the association between HIV status, mental health, medication toxicity, and other psychosocial factors with AET adherence among South African breast cancer survivors.

## Methods

### Setting and Participants

Participants were enrolled from the breast health clinic at the Chris Hani Baragwanath Academic Hospital (CHBAH) in Johannesburg, South Africa. CHBAH is the largest public hospital in South Africa and provides tertiary-level care to the residents of Soweto, a dense, predominantly black and socioeconomically diverse urban neighborhood of Johannesburg. At CHBAH, women with breast cancer have low- or no-cost access to breast surgery, chemotherapy, therapeutic radiation, adjuvant trastuzumab, and endocrine therapy. Approximately 25% of breast cancer patients treated at CHBAH have co-morbid HIV.[18]

For this study, we enrolled female patients with anatomic stage I to III, HR + breast cancer who had completed curative-intent breast surgery, had no evidence of current local or distant recurrence, and were currently being prescribed either tamoxifen or an aromatase inhibitor through the CHBAH breast clinic. As study procedures included six months of follow-up for adherence and use of extended adjuvant endocrine therapy is inconsistent in our setting, participants were ineligible if they had been prescribed endocrine therapy for longer than 4.5 years. To identify potentially eligible participants, we reviewed the medical records of women already enrolled in the South African Breast Cancer and HIV Outcomes (SABCHO) cohort study, which has been prospectively enrolling women with newly diagnosed breast cancer at CHBAH and four other public South African hospitals since 2015.[19] We attempted to contact potentially eligible participants identified on this pre-screen via telephone to offer study enrollment. In addition, eligible women who were newly starting endocrine therapy at the clinic were offered enrollment regardless of SABCHO participation.

### Study Procedures and Measures

Data were collected from participants at three time points that corresponded to their regularly scheduled breast clinic surveillance visits: an initial enrollment visit and two follow-up visits at 12- and 24-weeks post-enrollment. At the time of enrollment, we collected demographic data, breast cancer clinical data (*i.e.,* stage, grade, HER2 status, and type of endocrine therapy), and, if relevant, HIV clinical data (*i.e.,* infection status, timing of diagnosis, timing and type of anti-retroviral therapy, most recent CD4 count and HIV viral load). Household wealth data was taken from existing SABCHO records.

At enrollment, participants also completed four questionnaires measuring factors that might influence AET adherence. The Sources of Social Support Scale (SSSS) measured the degree of emotional and practical support participants received from different social relations.[20] The Beliefs About Medication Questionnaire-AET (BMQ-AET) captured their impression of AET’s importance to their long-term health and use of medications in general.[21] The Health Literacy Test for Limited Literacy populations (HELT-LL) measured functional health literacy.[22] The Communication and Attitudinal Self-Efficacy (CASE-Cancer) scale described self-reported capacity to maintain a positive attitude and communicate with providers when addressing health problems.[23]

During both their 12- and 24-week study visits, participants completed three questionnaires to quantify recent symptoms of depression, anxiety, and stress: the Center for Epidemiological Studies Depression Scale (CES-D), the Generalized Anxiety Disorder 7-Item Scale (GAD-7), and the Perceived Stress Scale (PSS).[24–26] At these visits, they also completed the Breast Cancer Prevention Trial Eight Symptom Scale Plus (BESS-Plus), measuring common AET-related musculoskeletal, vasomotor, gastrointestinal, gynecologic/sexual, bladder, weight, and fatigue/pain/malaise symptoms.[27]

All psychometric instruments were translated to isiZulu using the forwards-backwards translation approach and presented to patients in English and isiZulu. Participants had the option to have study staff read and record their responses to these questionnaires, and nearly all chose this approach. Starting in August 2022, we also introduced a series of custom visual cues for each questionnaire to illustrate directionality and strength of response choices. Participants who had been enrolled prior to the introduction of these visual scales repeated the SSSS, BMQ-AET, and CASE-Cancer questionnaires at their next follow-up visit.

AET adherence was measured in two ways. Self-reported adherence was captured by administering the Medication Adherence Report Scale, 5-item version (MARS-5) at 12 and 24 weeks.[28] Unfortunately, post-hoc analysis of MARS-5 showed nearly universal perfect adherence and was considered systemically unreliable. It was ultimately not used for analysis.

We also collected AET pill count data at each study visit to estimate the number of pills consumed over the study period. At enrollment, participants were instructed to no longer use any AET pills they had at home and were given a new prescription for three months’ worth of medication. They were then instructed to bring all remaining pills to their 12-week follow-up visit. At that follow-up visit, participants’ personal medical records were reviewed for documentation of medication refills, and remaining tablets were counted. Another prescription was given for 12 further weeks of medication and the process of counting pharmacy refills and remaining pills was repeated at 24 weeks. Of note, on August 16, 2022, in response to concern that some patients were continuing to take pills they had possessed prior to study enrollment, we began asking all participants to bring existing AET pills to their enrolment visit so that pre-study AET could be placed in a taped plastic bag as a physical reminder to only use newly prescribed pills while on study. Pill count data collection prior to this change in study procedure was excluded from analysis. An adherence ratio was calculated for each participant over each 12-week period by dividing the difference between collected and remaining pills by the exact number of days between visits. Study staff took detailed notes on any ambiguity or discrepancy in pill count data at the time of collection, and all data was hand reviewed by the authors. Instances with irresolvable count discrepancies were considered uninterpretable and excluded from analysis.

### Statistical Analysis

Participants’ demographic and breast cancer clinical data was described and compared by HIV infection status using Fishers exact and Wilcoxon rank sum tests. To account for many participants contributing two adherence ratio values (*i.e.,* at the separate 12- and 24-week visits), our crude analyses for association between AET adherence and individual clinical and psychosocial factors were performed used hierarchical linear models (HLM) of adherence ratio with only the relevant patient factor and study timepoint as covariates. To adjust for all evaluated clinical and psychometric factors’ impact on AET adherence, we developed a structural equation model (SEM) for adherence. Our *a priori* model included an HIV covariate and latent variables for healthcare savvy, social status, mental health, and medication burden. Again, in our structural model, each 12-week study interval for each participant was treated as an individual data point.

Overall fit of the model was determined by recommended criteria, including a comparative fit index (CFI) greater than .95 and root mean squared error of approximation (RMSEA) less than .06. In the case of poor model fit, modification indices guided model re-specification (e.g., inclusion of a residual covariance; removal of an indicator variable within a latent variable).[29] Once a final model was established, we repeated separate analyses on the sub-groups of participants with and without HIV to evaluate for differential associations between adherence and our latent variables in those groups.

## Results

Between April 26, 2022 and July 11, 2023, 810 women receiving breast cancer care at CHBAH were pre-screened for study eligibility, and 453 were deemed potentially eligible. Of those women, we were unable to contact 103 women, 69 women were contacted but declined study participation, and 280 were enrolled. Of these 280, 266 attended their 12-week visit. Twelve-week visit data was excluded for 115 women because they were enrolled prior to the addition of study procedures to prevent use of pre-existing AET supply, for 11 women because pill count data contained irreconcilable discrepancies, and for 12 women because their 12-week visit actually occurred <63 or >105 days from enrollment. Twelve-week data from the remaining 128 women was included for analysis. Of the 280 enrolled participants, 243 also attended their 24-week visit. Twenty-four-week visit data was excluded for 25 women because pill count data discrepancies could not be reconciled and for 1 woman whose visit occurred >105 days from enrollment. Twenty-four-week data from the remaining 217 women was included for analysis. In total, 239 participants contributed data at either 12 or 24 weeks, 63 (26.3%) of whom had comorbid HIV. The rate of HIV infection was not significantly different between participants contributing data and the rates in potentially eligible women who could not be reached (24.3%), who declined participation (20.3%), or who were enrolled but contributed no adherence data (17.1%).

Among all 239 participants, 116 (48.5%) had stage II cancer, 100 (41.8%) had stage III cancer, and 58 (24.3%) were HER2 over-expressing (Table 1). Tamoxifen was the more commonly used AET and prescribed to 191 (79.9%) of participants. The median age of participants with and without HIV infection differed: 47.4 years in women living with HIV and 57.7 years in those without. Participants with HIV also tended to be less wealthy than those without HIV.

Few differences were seen in the reported social support and demonstrated health literacy of participants with and without HIV infection (Table 2). Women with comorbid HIV did describe marginally higher care-related self-efficacy, reporting significantly increased participation in their own care and trends towards more positive attitude and information seeking behavior. Self-reports of depression, anxiety, and stress did not differ. Among AET-related side effects, women living with HIV experienced significantly more vasomotor symptoms and more dissatisfaction with their weight control.

The median AET adherence ratio was 0.88 in participants with HIV vs 0.89 in those without HIV (crude HLM HIV status β-coefficient (β) −0.03, standard error (SE) 0.03, p = 0.31) (Table 3). Adherence ratios were ≥ 80% in 52 of 96 (54.2%) 12-week time periods collected from participants with HIV and in 152 of 249 (61.0%) 12-week time periods from those without HIV (chi-square p = 0.24). We did find significant associations between decreased adherence ratio and greater specific concerns about using AET (β −0.01, SE 0.003, p = 0.001) and greater anxiety (β −0.01, SE 0.002, p = 0.04) (Table 3). Marginally significant associations were present between increased adherence and increasing wealth (β 0.02, SE 0.01, p = 0.06), increasing social support from friends (β 0.01, SE 0.004, p = 0.09), and decreased vasomotor symptoms (β −0.01, SE 0.003, p = 0.07).

**Figure 1:**
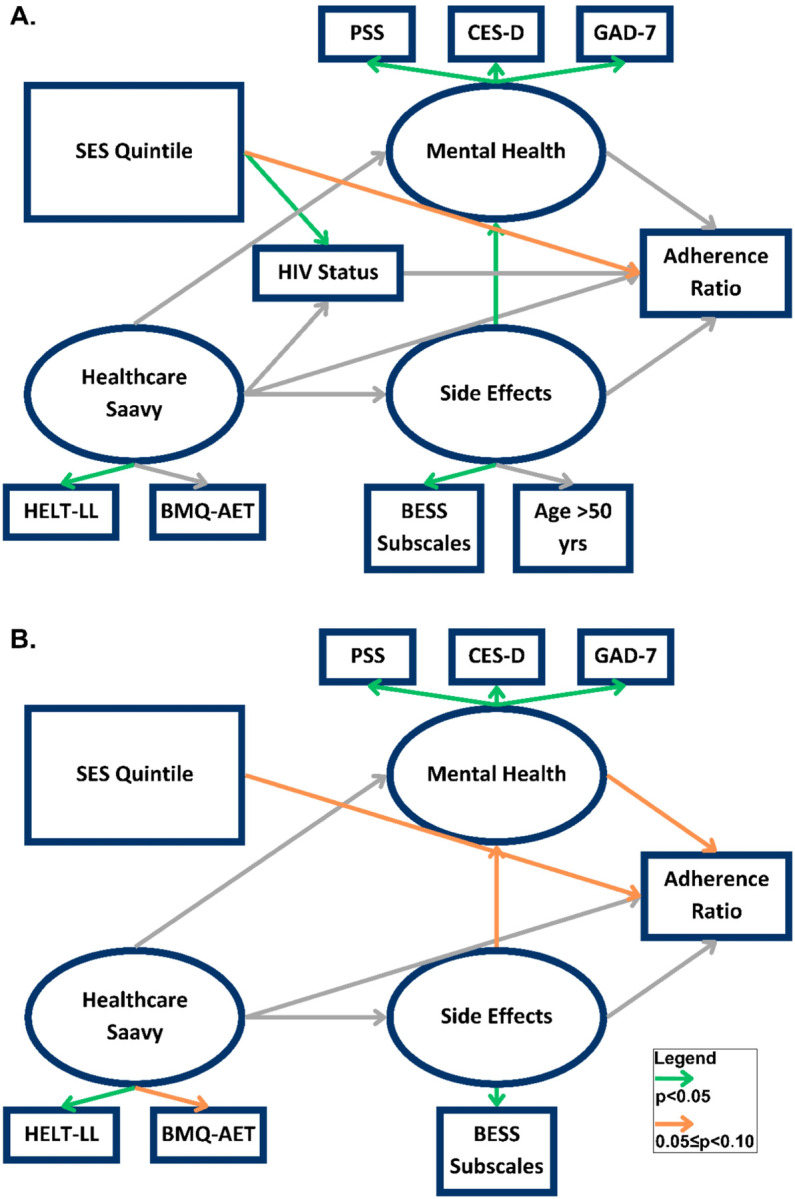
A. Final structural equation model of adjuvant endocrine therapy adherence for entire study cohort. B. Final structural equation model of adjuvant endocrine therapy adherence including only participants with a comorbid HIV infection.

The final SEM model achieved good fit (comparative fit index 0.97, root mean square error of approximation 90% confidence interval 0.02–0.05) (Fig. 1). HIV was still not associated with adherence ratio (β 0.01, SE 0.02, p = 0.71). The only latent variable demonstrating a trend towards association with adherence was wealth quintile (β 0.02, SE 0.01, p = 0.06). The AET side effects latent variable showed strong association with the mental health latent variable (β 1.4, SE 0.35, p < 0.001).

Using the same SEM in the subgroup of women with comorbid HIV infection, a trend towards association between wealth quintile and adherence remained (β 0.04, SE 0.02, p = 0.08) and a trend towards association between mental health and adherence emerged (β −0.02, SE 0.01, p = 0.10) (Fig. 1).

In the subgroup of women without comorbid HIV infection, neither wealth nor mental health showed any association with adherence.

## Discussion

In this prospective study of 239 South African women with early stage, hormone receptor positive breast cancer, daily adherence to AET was high in both women with comorbid HIV infection and those without: 88% and 89%, respectively. After adjusting for mental health symptoms, severity of AET-related adverse effects, and a variety of personal beliefs and social circumstances, there was no appreciable difference in adherence based on HIV infection. When specifically evaluating women with comorbid HIV, we did find trends toward a modest increase in adherence with increasing household wealth and a decrease in adherence with increasing mental health symptoms, though neither of these associations reached full statistical significance.

Medication adherence is difficult to measure even in high-income settings. Most published data from high-income countries relies on administrative data, such as pharmacy or insurance claims records, to estimate the ratio of pills obtained over some period of days, and medication coverage of ≥ 80% of days typically being considered adherent. Using these methods, AET adherence among various cohorts has been estimated at 33–89%, and data from the United States shows ~ 50% adherence as patients approach the end of five years of treatment.[11, 30] The proportion of women in this low-income cohort with adherence rates ≥ 80% was comparable to women in HICs, which at least assuages concern that this more vulnerable group of women is drastically less adherent than North American and European women. These findings are reminiscent of the literature on antiretroviral (ARV) research showing comparable or even superior adherence in sub-Saharan African versus North American patients.[31]

The existing literature on AET use has identified very old or young age, high medication costs and low SES status, AET side effects and low treatment satisfaction, depression, other comorbidities, poor social support, and perception of low recurrence risk or low AET efficacy as predictors of poor adherence in women from HICs.[10, 13, 14, 32] Our crude analyses of individual clinical and psychosocial factors did not replicate all of these findings, but less adherent patients did express greater concerns about using AET medications and greater anxiety in general. Statistical trends also pointed towards the possibility of decreased adherence in poorer women and women experiencing more vasomotor symptoms (*e.g.,* hot flashes). Those relationships are broadly consistent with research performed in North America and Europe and suggests that, while “dose-effect” might differ, the same types of predictors of adherence are relevant to women in South Africa.

Even among hormone receptor positive breast cancer patients who do not discontinue AET altogether, incomplete adherence is associated with 49% higher mortality.[9] However, our findings do not suggest that differences in AET adherence contribute to mortality disparities by HIV infection status seen in South African patients. Crude rates of adherence were practically identical between participants with and without HIV and the small difference in the proportions of women with adherence rates ≥ 80% was not statistically significant. Analysis of the SABCHO cohort data has previously demonstrated no significant differences based on HIV status in time to cancer treatment, chemotherapy dose intensity, or receipt of endocrine and radiation therapy.[33–35] This study provides further evidence that the causes of increased mortality among South African breast cancer patients with HIV go beyond differences in cancer treatment.

Our structural equation model’s associations of household wealth and mental health symptoms with adherence in just those breast cancer patients with comorbid HIV is hypothesis generating. In high-income settings, both interventions addressing medication costs and interventions with training in coping skills have shown potential for increasing AET adherence.[36, 37] Regarding mental health’s role, self-report of depression, anxiety, and acute stress did not differ based on HIV status. However, all three were universally prevalent, with the median participant reporting symptoms consistent with clinical depression and at least moderate stress and a quarter reporting at least moderate anxiety. If adherence-promoting, cognitive behavioral therapy-based interventions are to be adapted for women in LMICs, breast cancer patients living with HIV may be an appropriate group to target early for maximal effect.

Our findings should be considered within the context of the limitations of our study design and setting. High quality administrative data are rare in sub-Saharan Africa and is not available for describing AET adherence. The development of alternative approaches to measuring medication adherence in LMICs have largely been driven by HIV and antiretroviral research. Medication event monitoring systems (*e.g.,* electronic pill bottle caps or pill boxes that record when medication is accessed) were developed for HIV/ARV research and provide reliable, but expensive, data.[38] Pill counting, the adherence measure method used in this study, is more commonly used in LMICs, but is vulnerable to pill dumping (*i.e.,* intentional or accidental failure to bring all remaining pills to study visits for counting). In our study, participants typically possessed an existing supply of pills at the study start and had multiple options for obtaining refills between study visits. We worked to minimize mis-counting with telephone reminders to bring all pills to study visits, collection of all available prescription refill data, placing existing medications in a taped bags at study enrollment, and hand-reviewing all pill count data for apparent inconsistencies.

Pill counting relies on actively enrolled participants, while administrative data includes a wider range of study subjects. As such, our data largely excludes women who have ceased using AET altogether and captures adherence only for those still attending surveillance clinic visits. Most AET adherence data from HICs includes “non-persisting” women in estimates of non-adherence rates, so our values are not directly comparable.[30] Notably, the rate of HIV infection among this study’s enrolled participants was not significantly different from various types of eligible non-participants, so we do not expect that this limitation impacted our primary finding. It is also difficult to assess the extent to which participants’ modified their pill-taking behavior in response to simply being observed.

HIV viral load also provides an objective biomarker correlate for ARV adherence, while there is no widely available equivalent for checking the accuracy of AET adherences measurements. Specialty laboratories can measure tamoxifen serum concentrations via liquid chromatography-mass spectrometry. We previously used this approach to estimate tamoxifen adherence in a separate group of women with breast cancer at CHBAH and showed a lower overall rate of adherence (44%) and a doubling of poor adherence in women with comorbid HIV.[18] Unfortunately, that study did not have pill count data to correlate with serum concentrations, and the possibility of differences in tamoxifen metabolism could not be evaluated. Serum samples from our study’s cohort will allow for future testing of the relationship between pill count data and tamoxifen serum concentration.

In conclusion, we did not find a significant difference in AET adherence across South African breast cancer patients with and without HIV. Lower household wealth and greater mental health symptoms may be associated with reduced adherence in women with comorbid cancer and HIV, presenting potential targets for future interventions to improve AET adherence in this vulnerable group.

## Figures and Tables

**Table 1:** Enrolled Participant Characteristics by HIV Infection Status

	HIV Negative (N = 176)	HIV Positive (N = 63)	Total (N = 239)	p-value
**Age of Enrollment (median, IQR)**	57.7 (47.2–67.0)	47.4 (44.1–54.1)	53.7 (45.8–63.5)	<0.0001
**Anatomic Breast Cancer Stage (n, %)**				0.75
**I**	16 (9.1)	7 (11.1)	23 (9.6)	
**II**	84 (47.7)	32 (50.8)	116 (48.5)	
**III**	76 (43.2)	24 (38.1)	100 (41.8)	
**HER2 Status (n, %)**				0.77
**Negative**	120 (68.2)	43 (68.3)	163 (68.2)	
**Positive**	42 (23.9)	16 (25.4)	58 (24.3)	
**Equivocal**	11 (6.3)	4 (6.4)	15 (6.3)	
**Missing**	3 (1.7)	0 (0.0)	3 (1.3)	
**Adjuvant Endocrine Therapy (n,%)**				0.09
**Tamoxifen**	136 (77.3)	55 (87.3)	191 (79.9)	
**Aromatase Inhibitor**	40 (22.7)	8 (12.7)	48 (20.1)	
**Years since Breast Cancer Diagnosis (median, IQR)**	3.4 (2.0–4.2)	2.9 (1.2–3.9)	3.3 (1.8–4.1)	0.08
**Number of Other Medications Used (Non-ART) (median, IQR)**	1 (0–3)	0 (0–2)	1 (0–2)	0.04
**Married/Domestic Partner (n, %)**	88 (50.0)	28 (44.4)	116 (48.5)	0.49
**Tobacco Smoking (n, %)**	17 (9.7)	6 (9.5)	23 (9.7)	0.97
**Alcohol Use (n, %)**	36 (20.5)	20 (31.8)	56 (23.4)	0.07
**Wealth Percentile (n, %)**				0.0003
**< 20th (Lowest)**	12 (6.9)	6 (9.5)	18 (7.6)	
**20th to < 40th**	30 (17.2)	28 (44.4)	58 (24.5)	
**40th to < 60th**	46 (26.4)	8 (12.7)	54 (22.8)	
**60th to < 80th**	46 (26.4)	11 (17.5)	57 (24.1)	
**≥ 80th**	40 (23.0)	10 (15.9)	50 (21.1)	

Abbreviations: ART – antiretroviral therapy; HER2 - human epidermal growth factor receptor 2

**Table 2:** Questionnaire Responses by HIV Infection Status

Instrument	HIV Negative (N = 176)	HIV Positive (N = 63)	p-value
Median (IQR)			
**SSSS (range: 0–4)**			
**Partner**			
**Emotional Support**	3.8 (3.0–4.0)	3.7 (2.8–4.0)	0.81
**Negative Support**	4.0 (4.0–4.0)	4.0 (4.0–4.0)	0.28
**Informational Support**	2.5 (0.0–4.0)	2.0 (0.0–4.0)	0.55
**Instrumental Support**	4.0 (2.0–4.0)	4.0 (1.0–4.0)	0.49
**Women in Your Family**			
**Emotional Support**	4.0 (3.3–4.0)	4.0 (3.7–4.0)	0.11
**Negative Support**	4.0 (4.0–4.0)	4.0 (4.0–4.0)	0.31
**Informational Support**	4.0 (2.0–4.0)	4.0 (2.0–4.0)	0.59
**Instrumental Support**	4.0 (2.0–4.0)	4.0 (2.0–4.0)	0.26
**Friends**			
**Emotional Support**	4.0 (3.3–4.0)	3.7 (3.3–4.0)	0.26
**Negative Support**	4.0 (4.0–4.0)	4.0 (4.0–4.0)	0.03
**Informational Support**	4.0 (2.0–4.0)	3.0 (1.0–4.0)	0.22
**Instrumental Support**	4.0 (2.0–4.0)	3.0 (1.0–4.0)	0.21
**Healthcare Provider**			
**Emotional Support**	4.0 (3.5–4.0)	4.0 (4.0–4.0)	0.02
**Negative Support**	4.0 (4.0–4.0)	4.0 (4.0–4.0)	0.97
**Informational Support**	4.0 (4.0–4.0)	4.0 (4.0–4.0)	0.07
**Instrumental Support**	4.0 (4.0–4.0)	4.0 (4.0–4.0)	0.86
**HELT-LL**			
**Functional Literacy (range: 0–14)**	9.0 (7.0–11.0)	9.0 (6.0–11.0)	0.70
**Communicative Literacy (range: 0–4)**	2.0 (1.0–3.0)	2.0 (1.0–4.0)	0.92
**Critical Literacy (range: 0–6)**	4.0 (2.0–4.0)	4.0 (2.0–6.0)	0.31
**BMQ**			
**AET Concerns (range: 0–20)**	4.0 (0.0–8.0)	5.0 (0.0–9.0)	0.39
**AET Necessity (range: 0–20)**	18.0 (15.0–20.0)	17.0 (16.0–20.0)	0.82
**General Overuse (range: 0–16)**	7.0 (4.0–10.0)	8.0 (4.0–10.0)	0.52
**General Harms (range: 0–16)**	2.0 (0.0–5.0)	3.0 (0.0–5.0)	0.73
**CASE-Cancer**			
**Participation in Care (range: 4–16)**	16.0 (14.0–16.0)	16.0 (16.0–16.0)	0.02
**Positive Attitude (range: 4–16)**	16.0 (15.0–16.0)	16.0 (16.0–16.0)	0.08
**Seeking Information (range: 4–16)**	16.0 (15.0–16.0)	16.0 (16.0–16.0)	0.07
**Mental Health Questionnaires (Mean score)**			
**CES-D (range: 0–60)**	15.5 (8.0–23.0)	17.0 (10.5–27.0)	0.16
**GAD-7 (range: 0–21)**	5.0 (2.0–9.0)	7.0 (3.0–11.0)	0.11
**PSS (range: 0–40)**	16.0 (12.0–21.0)	17.5 (13.0–23.0)	0.29
**AET-Related Side Effect Scores**			
**Cognitive (range: 0–12)**	4.0 (1.5–6.5)	4.0 (1.5–6.5)	0.62
**Musculoskeletal (range: 0–12)**	5.0 (2.5–8.0)	5.0 (2.5–8.0)	0.96
**Vasomotor (range: 0–12)**	2.0 (0.5–6.0)	5.0 (1.5–8.0)	0.01
**Gastrointestinal (range: 0–12)**	0.0 (0.0–0.5)	0.0 (0.0–1.0)	0.15
**Dyspareunia (range: 0–8)**	0.0 (0.0–1.0)	0.5 (0.0–1.5)	0.12
**Weight Control (range: 0–8)**	1.0 (0.0–3.0)	2.0 (1.0–3.5)	0.02
**Gynecologic (range: 0–12)**	0.5 (0.0–1.0)	0.5 (0.0–2.0)	0.34
**Urinary incontinence (range: 0–8)**	0.0 (0.0–1.3)	0.0 (0.0–1.0)	0.39
**Pain/Fatigue (range: 0–36)**	8.8 (5.0–13.0)	10.0 (6.0–13.0)	0.32

Abbreviations: AET – adjuvant endocrine therapy; BMQ – Beliefs About Medication Questionnaire; CASE-Cancer – Communication and Attitudinal Self-Efficacy scale; CES-D – Center for Epidemiological Studies Depression Scale; GAD-7 – Generalized Anxiety Disorder 7-Item Scale; HELT-LL – Health Literacy Test for Limited Literacy populations; PSS – Perceived Stress Scale; SSSS –Sources of Social Support Scale

**Table 3:** Crude Associations Between Clinical and Psychosocial Factors and AET Adherence Ratio

Factor	β-coefficient	Standard Error	p-value^1^
**Clinical**			
**Comorbid HIV Infection**	−0.03	0.03	0.31
**Age**	0.01	0.01	0.29
**BC Stage (I/II vs III)**	−0.01	0.03	0.64
**HER2 Status (Positive vs Negative)**	0.01	0.03	0.91
**Tamoxifen Prescription**	0.02	0.03	0.61
**Number of Other Medications Used (Non-ART)**	−0.01	0.01	0.42
**Social**			
**Married/Domestic Partner**	0.02	0.02	0.28
**Tobacco Smoking**	0.06	0.05	0.21
**Alcohol Use**	0.02	0.02	0.57
**Wealth Percentile**	0.02	0.01	0.06
**Psychosocial**			
**SSSS - Partner**	0.001	0.01	0.69
**SSSS - Women in Your Family**	0.002	0.01	0.63
**SSSS - Friends**	0.01	0.004	0.09
**SSSS - HCP**	0.002	0.01	0.70
**HELT-LL - Total Score**	0.003	0.003	0.32
**BMQ - AET Concerns**	−0.01	0.003	0.001
**BMQ - AET Necessity**	−0.003	0.004	0.40
**BMQ - General Overuse**	−0.01	0.003	0.11
**BMQ - General Harms**	−0.01	0.004	0.16
**CASE-Cancer - Total Score**	0.003	0.003	0.45
**CES-D Score**	−0.002	0.001	0.12
**GAD-7 Score**	−0.01	0.002	0.04
**PSS Score**	−0.002	0.002	0.29
**AET-Related Side Effects**			
**Cognitive**	−0.001	0.001	0.91
**Musculoskeletal**	−0.001	0.001	0.88
**Vasomotor**	−0.01	0.003	0.07
**Gastrointestinal**	−0.02	0.01	0.09
**Dyspareunia**	0.001	0.007	0.87
**Weight Control**	−0.001	0.006	0.84
**Gynecologic**	−0.001	0.001	0.91
**Urinary Incontinence**	−0.001	0.001	0.90
**Pain/Fatigue**	−0.001	0.002	0.57

1: P-value of the relevant factor in a hierarchical linear models of adherence ratio with study timepoint as the only other covariate,

Abbreviations: AET – adjuvant endocrine therapy; ART – antiretroviral therapy; BC – breast cancer; BMQ – Beliefs About Medication Questionnaire; CASE-Cancer – Communication and Attitudinal Self-Efficacy scale; CES-D – Center for Epidemiological Studies Depression Scale; GAD-7 – Generalized Anxiety Disorder 7-Item Scale; HELT-LL – Health Literacy Test for Limited Literacy populations; HER2 -human epidermal growth factor receptor 2; PSS – Perceived Stress Scale; SSSS – Sources of Social Support Scale

## Data Availability

De-identified data from this study is available upon request to Dr. Joffe to the fullest extent possible under South African law.

## References

[1] Coghill AE, Shiels MS, Suneja G, Engels EA (2015) Elevated Cancer-Specific Mortality Among HIV-Infected Patients in the United States. J Clin Oncol 33:2376–2383. 10.1200/JC0.2014.59.5967 26077242 PMC4500831

[2] Coghill AE, Suneja G, Rositch AF, (2019) HIV Infection, Cancer Treatment Regimens, and Cancer Outcomes Among Elderly Adults in the United States. JAMA Oncol. 10.1001/jamaoncol.2019.1742 PMC6681563 31369037

[3] Coghill AE, Han X, Suneja G, (2019) Advanced stage at diagnosis and elevated mortality among US patients with cancer infected with HIV in the National Cancer Data Base. Cancer 125:2868–2876. 10.1002/cncr.32158 31050361 PMC6663596

[4] Ayeni OA, O’Neil DS, Pumpalova YS, Impact of HIV infection on survival among women with stage I-III breast cancer: Results from the South African breast cancer and HIV outcomes study. International Journal of Cancer n/a: 10.1002/ijc.33981 PMC9133061 35218568

[5] McCormack VA, Febvey-Combes O, Ginsburg O, dos-Santos-Silva I (2018) Breast cancer in women living with HIV: A first global estimate. International Journal of Cancer 143:2732–2740. 10.1002/ijc.31722 29992553

[6] O’Neil DS, Chen WC, Ayeni O, (2019) Breast Cancer Care Quality in South Africa’s Public Health System: An Evaluation Using American Society of Clinical Oncology/National Quality Forum Measures. JGO 1–16. 10.1200/JGO.19.00171 PMC6882520 31770052

[7] Effects of chemotherapy and hormonal therapy for early breast cancer on recurrence and 15-year survival: an overview of the randomised trials. The Lancet 365:1687–1717. 10.1016/S0140-6736(05)66544-0 15894097

[8] Relevance of breast cancer hormone receptors and other factors to the efficacy of adjuvant tamoxifen: patient-level meta-analysis of randomised trials. The Lancet 378:771–784. 10.1016/S0140-6736(11)60993-8 PMC3163848 21802721

[9] Hershman DL, Shao T, Kushi LH, (2011) Early discontinuation and non-adherence to adjuvant hormonal therapy are associated with increased mortality in women with breast cancer. Breast Cancer Res Treat 126:529–537. 10.1007/s10549-010-1132-4 20803066 PMC3462663

[10] Chlebowski RT, Kim J, Haque R (2014) Adherence to Endocrine Therapy in Breast Cancer Adjuvant and Prevention Settings. Cancer Prevention Research 7:378–387. 10.1158/1940-6207.CAPR-13-0389 24441675 PMC11649036

[11] Hershman DL, Kushi LH, Shao T, (2010) Early Discontinuation and Nonadherence to Adjuvant Hormonal Therapy in a Cohort of 8,769 Early-Stage Breast Cancer Patients. Journal of Clinical Oncology 28:4120–4128. 10.1200/JC0.2009.25.9655 20585090 PMC2953970

[12] Demissie S, Silliman RA, Lash TL (2001) Adjuvant tamoxifen: predictors of use, side effects, and discontinuation in older women. J Clin Oncol 19:322–328. 10.1200/JC0.2001.19.2.322 11208822

[13] Hershman DL, Kushi LH, Hillyer GC, (2016) Psychosocial factors related to non-persistence with adjuvant endocrine therapy among women with breast cancer: the Breast Cancer Quality of Care Study (BQUAL). Breast Cancer Research and Treatment 157:133–143. 10.1007/s10549-016-3788-x 27086286 PMC4867255

[14] Neugut AI, Hillyer GC, Kushi LH, (2012) Non-initiation of adjuvant hormonal therapy in women with hormone receptor-positive breast cancer: The Breast Cancer Quality of Care Study (BQUAL). Breast Cancer Research and Treatment 134:419–428. 10.1007/s10549-012-2066-9 22527111 PMC3561459

[15] Paranjpe R, John G, Trivedi M, Abughosh S (2019) Identifying adherence barriers to oral endocrine therapy among breast cancer survivors. Breast Cancer Research and Treatment 174:297–305. 10.1007/s10549-018-05073-z 30523459

[16] Roberts MC, Wheeler SB, Reeder-Hayes K (2015) Racial/Ethnic and Socioeconomic Disparities in Endocrine Therapy Adherence in Breast Cancer: A Systematic Review. American Journal of Public Health 105:e4–e15. 10.2105/AJPH.2014.302490 PMC4455526 25905855

[17] Ayeni OA, Chiwambutsa S, Chen WC, (2023) The impact of HIV on non-adherence for tamoxifen among women with breast cancer in South Africa. Breast Cancer Res Treat 197:647–659. 10.1007/s10549-022-06835-6 36538247 PMC10149344

[18] Ayeni OA, O’Neil DS, Pumpalova YS, (2022) Impact of HIV infection on survival among women with stage I-III breast cancer: Results from the South African breast cancer and HIV outcomes study. Int J Cancer 151:209–221. 10.1002/ijc.33981 35218568 PMC9133061

[19] Cubasch H, Ruff P Joffe M, (2016) South African Breast Cancer and HIV Outcomes Study: Methods and Baseline Assessment. JGO 3:114–124. 10.1200/JGO.2015.002675 PMC5493271 28706996

[20] Kinsinger SW, Laurenceau J-P, Carver CS, Antoni MH (2011) Perceived partner support and psychosexual adjustment to breast cancer. Psychology & Health 26:1571–1588. 10.1080/08870446.2010.533771 21598184

[21] Brett J, Hulbert-Williams NJ, Fenlon D, (2017) Psychometric properties of the Beliefs about Medicine Questionnaire-adjuvant endocrine therapy (BMQ-AET) for women taking AETs following early-stage breast cancer. Health Psychol Open 4:. 10.1177/2055102917740469 PMC5779943 29379627

[22] Marimwe C, Dowse R (2019) Health literacy test for limited literacy populations (HELT-LL): Validation in South Africa. Cogent Medicine 6:1650417. 10.1080/2331205X.2019.1650417

[23] Wolf MS, Chang C-H, Davis T, Makoul G (2005) Development and validation of the Communication and Attitudinal Self-Efficacy scale for cancer (CASE-cancer). Patient Educ Couns 57:333–341. 10.1016/j.pec.2004.09.005 15893217

[24] Radloff LS (2016) The CES-D Scale: A Self-Report Depression Scale for Research in the General Population. Applied Psychological Measurement. 10.1177/014662167700100306

[25] Spitzer RL, Kroenke K, Williams JBW, Lowe B (2006) A brief measure for assessing generalized anxiety disorder: the GAD-7. Arch Intern Med 166:1092–1097. 10.1001/archinte.166.10.1092 16717171

[26] Cohen S, Kamarck T, Mermelstein R (1983) A global measure of perceived stress. J Health Soc Behav 24:385–396 6668417

[27] Stanton AL, Bernaards CA, Ganz PA (2005) The BCPT Symptom Scales: A Measure of Physical Symptoms for Women Diagnosed With or at Risk for Breast Cancer. JNCI: Journal of the National Cancer Institute 97:448–456. 10.1093/jnci/dji069 15770009

[28] Chan AHY, Horne R, Hankins M, Chisari C (2020) The Medication Adherence Report Scale: A measurement tool for eliciting patients’ reports of nonadherence. Br J Clin Pharmacol 86:1281–1288. 10.1111/bcp.14193 31823381 PMC7319010

[29] Hu L, Bentler PM (1999) Cutoff criteria for fit indexes in covariance structure analysis: Conventional criteria versus new alternatives. Structural Equation Modeling: A Multidisciplinary Journal 6:1–55. 10.1080/10705519909540118

[30] Yussof I, Mohd Tahir NA, Hatah E, Mohamed Shah N (2022) Factors influencing five-year adherence to adjuvant endocrine therapy in breast cancer patients: A systematic review. The Breast 62:22–35. 10.1016/j.breast.2022.01.012 35121501 PMC8818734

[31] Mills EJ, Nachega JB, Buchan I, (2006) Adherence to antiretroviral therapy in sub-Saharan Africa and North America: a meta-analysis. Jama 296:679–90. 10.1001/jama.296.6.679 16896111

[32] Cluze C, Rey D, Huiart L, (2012) Adjuvant endocrine therapy with tamoxifen in young women with breast cancer: determinants of interruptions vary over time. Annals of Oncology 23:882–890. 10.1093/annonc/mdr330 21788360

[33] Pumpalova YS, Ayeni OA, Chen WC, (2022) The Impact of Breast Cancer Treatment Delays on Survival Among South African Women. Oncologist 27:e233–e243. 10.1093/oncolo/oyab054 35274708 PMC8914482

[34] O’Neil DS, Ayeni OA, Farrow HA, (2023) The Impact of HIV infection on Neoadjuvant and Adjuvant Chemotherapy Relative Dose Intensity in South African Breast Cancer Patients. The Oncologist10.1093/oncolo/oyad056 PMC10546819 36943395

[35] O’Neil DS, Chen WC, Ayeni O, (2019) Breast Cancer Care Quality in South Africa’s Public Health System: An Evaluation Using American Society of Clinical Oncology/National Quality Forum Measures. J Glob Oncol 5:JGO.19.00171. 10.1200/JGO.19.00171 PMC6882520 31770052

[36] Bright EE, Finkelstein LB, Nealis MS, (2023) A Systematic Review and Meta-Analysis of Interventions to Promote Adjuvant Endocrine Therapy Adherence Among Breast Cancer Survivors. JCO 41:4548–4561. 10.1200/JCO.23.00697 PMC10553065 37531593

[37] Ream ME, Walsh EA, Jacobs JM, (2021) Brief relaxation training is associated with long-term endocrine therapy adherence among women with breast cancer: post hoc analysis of a randomized controlled trial. Breast Cancer Res Treat 190:79–88. 10.1007/s10549-021-06361-x 34410568 PMC9245682

[38] Castillo-Mancilla JR, Haberer JE (2018) Adherence Measurements in HIV: New Advancements in Pharmacologic Methods and Real-Time Monitoring. Curr HIV/AIDS Rep 15:49–59. 10.1007/s11904-018-0377-0 29380227 PMC5876155

